# Supplementary material for: E2F-dependent transcription determines replication capacity and S phase length
Source: Nat Commun. 2020 Jul 14;11:3503. doi: 10.1038/s41467-020-17146-z (PMC7360579; doi:10.1038/s41467-020-17146-z)
Supplement: Supplementary file 1 — Supplementary Information [file 41467_2020_17146_MOESM1_ESM.pdf]

Supplementary Information.

E2F-dependent transcription determines replication capacity and S phase length

Pennycook et al.

Supplementary Figure 1

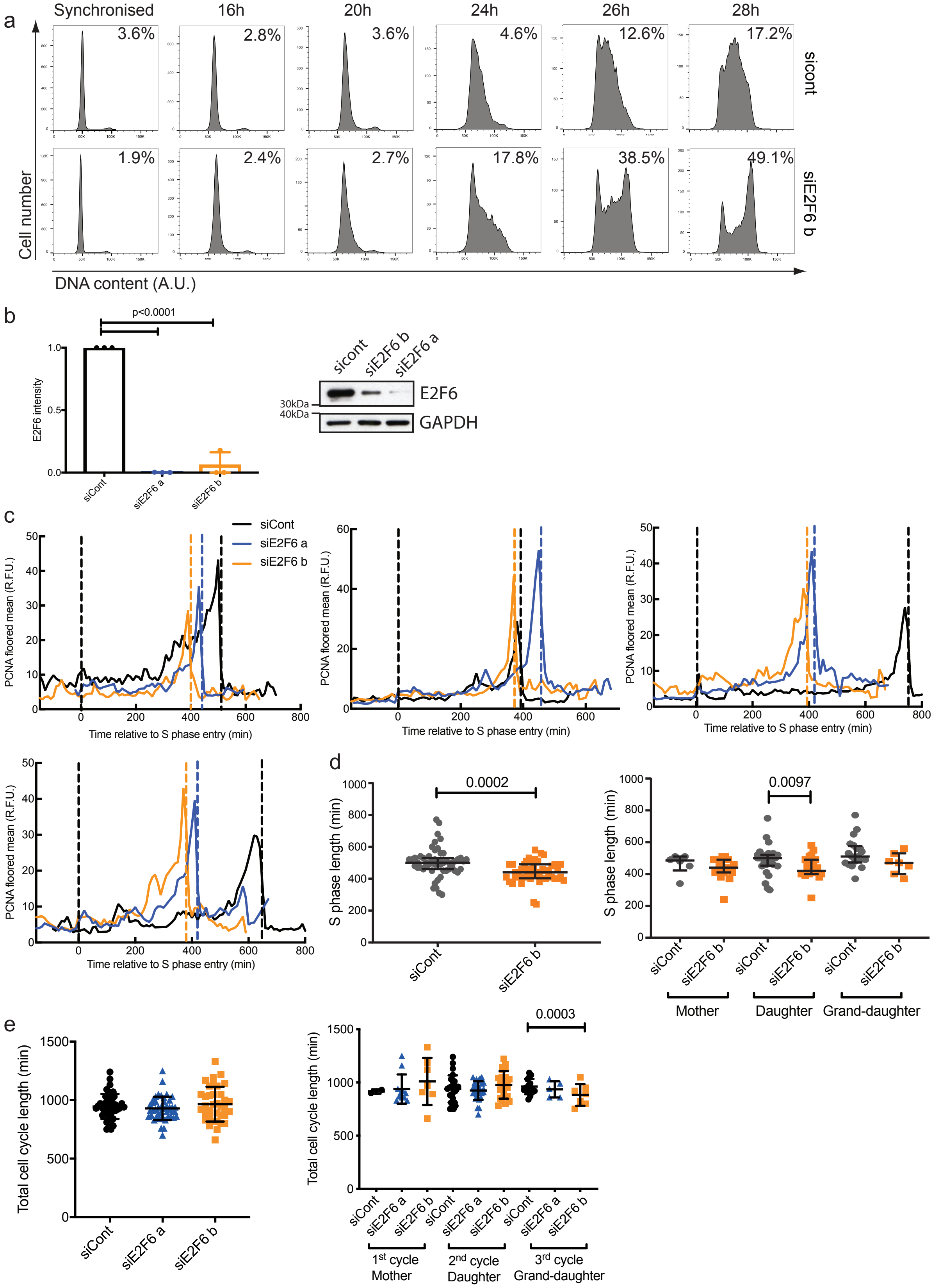

Supplementary Figure 1. E2F6 knockdown decreases S phase length. T98G cells were transfected and synchronised in G1 phase by serum starvation, starved cells were re-transfected and released in serum. (**a**) DNA was quantified by flow cytometry at the indicated times following release of cells into serum and transfection with control or E2F6 b siRNA. Nocodazole was added at 16 h post release at 100 ng/ml. Inset percentage reflects cells in G2/M phase based on DNA staining. Representative plots from n=3 experiments. (**b**) Whole cell extract from asynchronous RPE1 hTERT cells was collected 24 h following transfection with the indicated siRNAs and levels of E2F6 quantified using western blot. Representative western blot is shown. Measured values were normalised to GAPDH loading control and siCont, n=3 mean and S.D. shown. (**c**) Representative single traces of mRuby-PCNA levels (floored mean) over time in single RPE1 hTERT cells expressing p21-GFP and mRuby-PCNA and treated with the indicated siRNA. (**d**) Mean and S.D. S phase length in single RPE1 cells plotted as an average across 3 cell cycles, left, and within each imaged cycle, right. siCont n=55 cells (n=6, 18, 19 mother, daughter and grand-daughter cells, respectively), siE2F6 b n=56 (n=20, 7, 6). (**e)** Mean cell cycle length in single cells plotted as an average across 3 cell cycles, left, and within each imaged cycle, right. siCont n=42 cells (2, 7, 11 mother, daughter and grand-daughter cells, respectively), siE2F6 a n=36 (25, 22, 28), siE2F6 b n=44 (15, 7, 5). Mean with SD is shown. Significance was determined using a one-way ANOVA **(a)**, Kruskal-Wallis test **(d,e)**.

Supplementary Figure 2

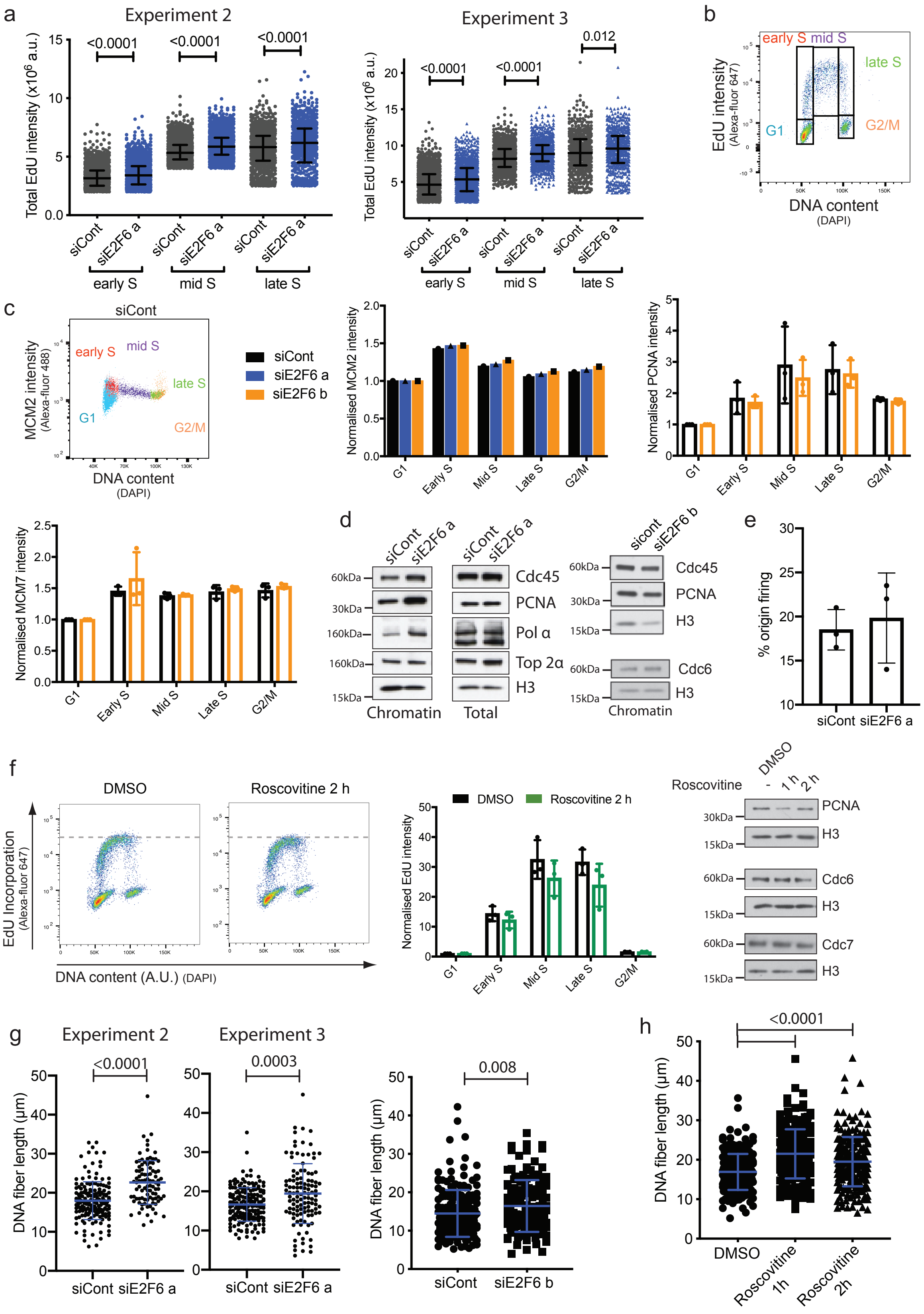

Supplementary Figure 2. Maintained E2F activity during S phase using an alternative siRNA increases cellular replication capacity (**a**) Asynchronous RPE1 cells were reverse transfected and 24h later pulsed with EdU for 30 min before fixation and immunofluorescence staining. Left, cell population was divided into 5 groups based on DNA stain and EdU incorporation, as indicated on the coloured scatter plots. G1: EdU negative and G1 DNA content, Early S: EdU positive and G1 DNA content, Mid S: EdU positive and intermediate DNA content, Late S: EdU positive and G2 DNA content. Mean and S.D. of 2 biological repeats are shown. **(b)** FACS gating strategy for cell cycle phase grouping based on EdU and DAPI staining. **(c)** PCNA, MCM2 and MCM7 content on chromatin were quantified by flow cytometry 24 h following transfection with the indicated siRNAs. G1: EdU negative and G1 DNA content, Early S: EdU positive and G1 DNA content, Mid S: EdU positive and intermediate DNA content, Late S: EdU positive and G2 DNA content, G2: EdU negative and G2 DNA content. PCNA and MCM7 n=3 mean and S.D. shown, MCM2 n=1. Values are normalised to G1. (**d**) Western blot of whole cell extract and chromatin fraction of RPE1 hTERT cells 24 h following transfection with the indicated siRNAs. Histone H3 as loading control. Representative of n=3 experimental repeats **(e)** Samples for DNA fibre analysis were collected 24h following transfection of RPE1 hTERT cells with the indicated siRNAs. Origin firing frequency was calculated from n=4 experimental repeats. (**f**) RPE1 cells were treated with CDK inhibitor 25µM Roscovitine and pulse labelled with EdU for 2 h prior to collection. DNA content and EdU incorporation was analysed by flow cytometry. Representative flow cytometry plot shown, with mean and S.D. quantification from n=3 experiments. Right, chromatin-bound protein analysed by western blot, Histone H3 is used as a loading control. (**g**) DNA fibres were collected from RPE1 hTERT cells 24 h following transfection with the indicated siRNAs. Dot plots are from one experimental repeat each, mean and S.D. shown. (**h**) RPE1 hTERT cells were treated with Roscovitine prior to DNA fibre analysis. Mean and S.D. from n=3 experiments shown, at least 150 fibres were counted per condition. Significance was determined by a two-tailed Mann-Whitney test **(a,g,h)**, 2-way ANOVA with Sidak's multiple comparisons test within each population **(c,f)**.

Supplementary figure 3

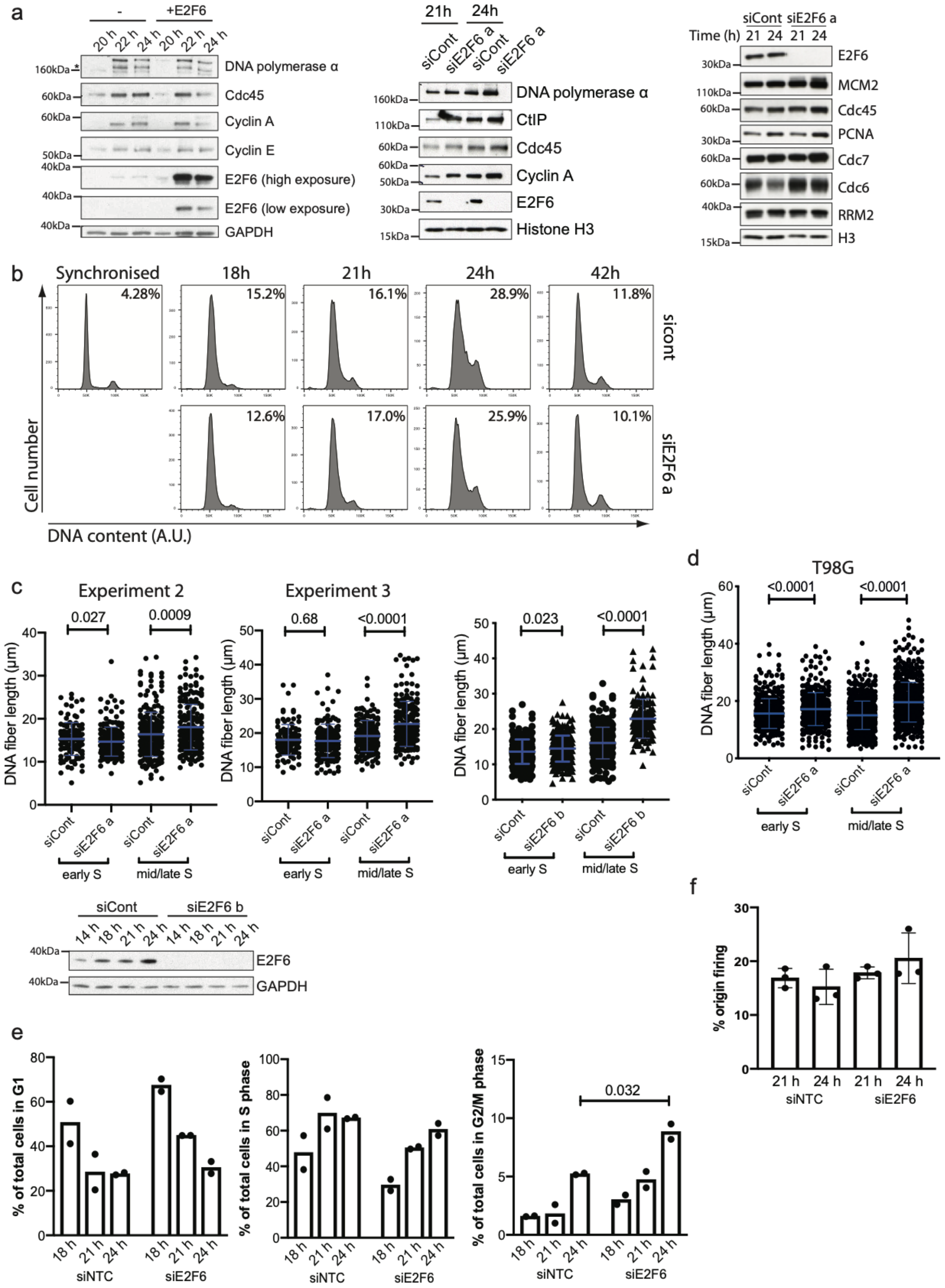

Supplementary Figure 3. Modulation of E2F-dependent transcription alters replication dynamics during S phase. (**a**) RPE1 cells were synchronised by contact inhibition and whole cell extracts from RPE1 hTERT cells (left) and RPE1 E2F6 cells (right) were collected at the indicated times following release. Left, cells were transfected with the indicated siRNAs following release. Right, 4 μg/ml doxycycline was added to cells 18 h following release to induce E2F6 overexpression. Representative of n=3 experimental repeats. * indicates a specific band. **(b)** RPE1 hTERT cells were synchronised by contact inhibition, transfected upon release and collected for flow cytometry analysis of DNA content at the indicated times after release. Data from one of n=3 independent experiments shown. Inset percentage reflects cells with 4N DNA content. **(c)** Cells were treated as in (b) and collected for DNA fibre analysis at early (20 h) and mid/late (25 h) S phase, quantification with mean and S.D. values of fibre lengths from n=2 experimental repeats are shown for siE2F6 a, one of n=3 independent experiments are shown for siE2F6 b, at least 200 fibres were counted per condition. Right, western blot of whole cell extract at the indicated times following release. GAPDH as loading control. (**d)** T98G cells were synchronised by serum starvation for 48 h, samples were collected for DNA fibre analysis at 22 h (early S phase) and 26 h (mid/late S phase) following release and transfection with the indicated siRNAs. Representative of n=2 experimental repeats shown with mean and S.D.. **(e)** RPE1 cells were treated as in **b** and were pulse labelled with EdU before fixation for immunofluorescence analysis at the indicated timepoints following release. The percentage of cells in each phase of the cell cycle was determined by DNA (Hoechst) and EdU staining. Only significant differences between control and siE2F6 samples are shown, n=2 experimental repeats. **(f)** Origin firing quantified as the number of firing events as a percentage of total fibres counted n=3 experimental repeats, mean and S.D. shown. Significance within each timepoint was determined by a two-tailed Mann-Whitney test **(c,d)**, or a two-tailed student's t-test **(e,f)**.

Supplementary figure 4

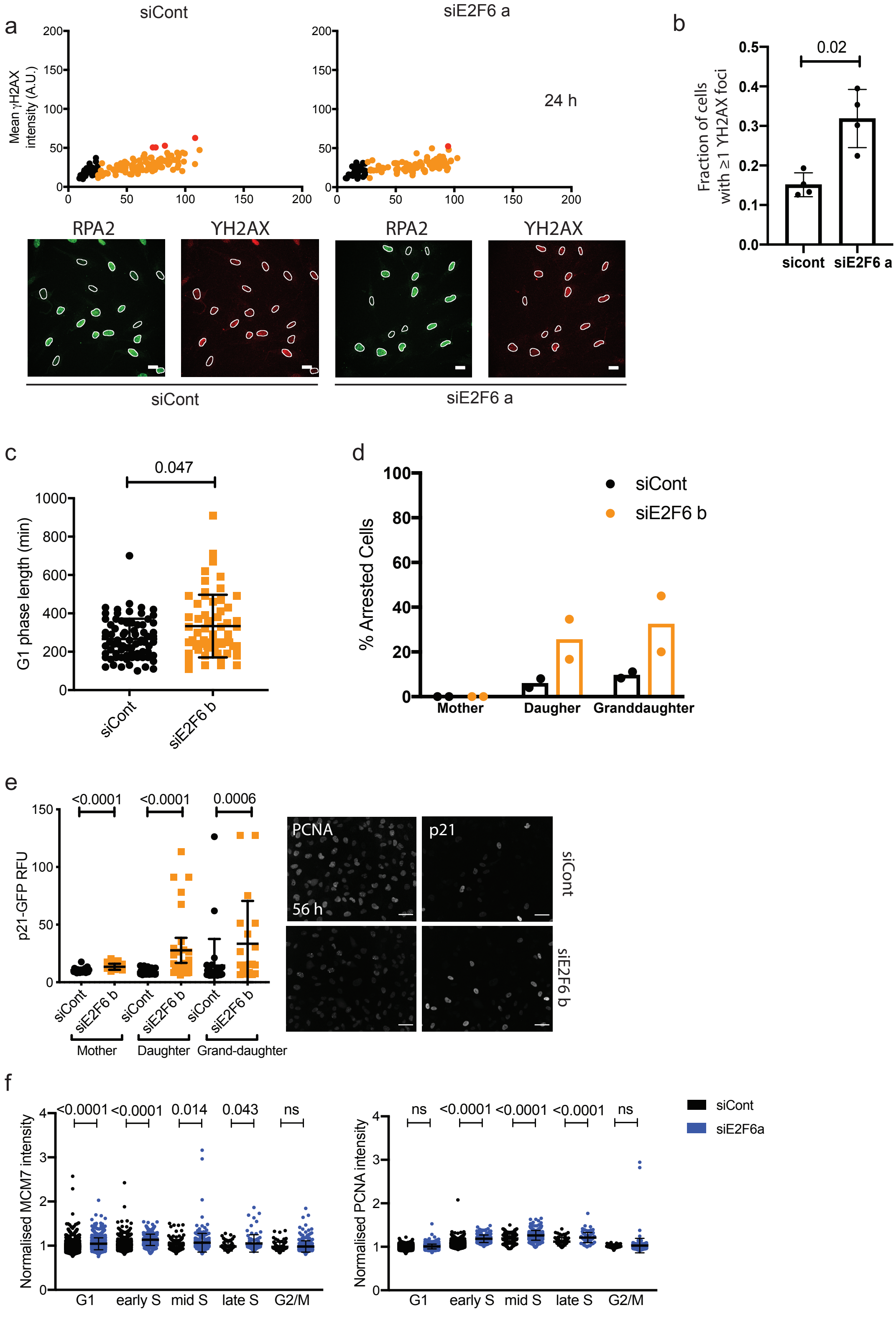

Supplementary Figure 4. **Increased E2F-dependent transcription in S phase does not cause in DNA damage during the first S phase. (a)** RPE1 cells were synchronised by contact inhibition and levels of chromatin-bound RPA2 and γH2AX were quantified by immunofluorescence mid/late S phase (24 h) of the first cell cycle after release. Black, non-S phase cells (RPA2 <50 A.U.); orange and red dots, low and high levels of γH2AX, respectively (arbitrary γH2AX threshold = 50 A.U.). Data is representative of three independent experiments, scale bar represents 20 µm. **(b)** Cells were synchronised as in (a) and the number of RPA negative cells with one or more γH2AX foci in the second cell cycle following release was quantified. At least 2000 cells scored per n=4 experiments, mean and S.D. shown. **(c)** Length of G1 phase calculated from live imaging of RPE1 mRuby-PCNA p21-GFP. siCont n=75, siE2F6 b n=57. Mean and S.D. shown. **(d)** Percentage of arrested and dividing cells were quantified from live imaging of RPE1 mRuby-PCNA p21-GFP cells through two divisions. Mean from two independent experiments shown. **(e)** Quantification of p21 levels in RPE1 mRuby-PCNA p21-GFP cells through two divisions. Images of p21 fluorescence intensity 56 h after commencement of imaging, scale bar =50 µm. Median with 95% confidence intervals are shown. siCont n=22,22,25 mother, daughter and grand-daughter cells, respectively, siE2F6 b n=31,20,28. **(f)** RPE1 mRubyPCNA cells were synchronised by contact inhibition and transfected upon release. 41 h following release cells were pulse labelled with EdU 30 min before fixation and immunofluorescence staining for MCM7 (left) and mRubyPCNA. Cell population was divided into 5 groups based on DNA stain and EdU incorporation as in Fig 2. Data with mean and S.D. from two experimental repeats are shown, normalised to the mean value of the NTC G1 population within each repeat. Significance was determined by two-tailed Mann-Whitney test for each marker compared to control at each timepoint **(a,e)**, two-tailed student's t-test **(b)**, Kruskal-Wallis test with Dunn's multiple comparisons test **(c)**, one-way ANOVA with Sidak's multiple comparison test **(f)**.
