# Supplementary material for: E2F-dependent transcription determines replication capacity and S phase length
Source: Nat Commun. 2020 Jul 14;11:3503. doi: 10.1038/s41467-020-17146-z (PMC7360579; doi:10.1038/s41467-020-17146-z)
Supplement: Supplementary file 3 — Reporting Summary [file 41467_2020_17146_MOESM3_ESM.pdf]

nature research

Corresponding author(s): *Double-blind peer review submissions: write DBPR and your manuscript number here instead of author names.*

Last updated by author(s): *YYYY-MM-DD*

Reporting Summary

Nature Research wishes to improve the reproducibility of the work that we publish. This form provides structure for consistency and transparency in reporting. For further information on Nature Research policies, see Authors & Referees and the Editorial Policy Checklist.

Statistics

For all statistical analyses, confirm that the following items are present in the figure legend, table legend, main text, or Methods section.

n/a	Confirmed	
☐	☒	The exact sample size (*n*) for each experimental group/condition, given as a discrete number and unit of measurement
☐	☒	A statement on whether measurements were taken from distinct samples or whether the same sample was measured repeatedly
☐	☒	The statistical test(s) used AND whether they are one- or two-sided<br>*Only common tests should be described solely by name; describe more complex techniques in the Methods section.*
☒	☐	A description of all covariates tested
☐	☒	A description of any assumptions or corrections, such as tests of normality and adjustment for multiple comparisons
☐	☒	A full description of the statistical parameters including central tendency (e.g. means) or other basic estimates (e.g. regression coefficient) AND variation (e.g. standard deviation) or associated estimates of uncertainty (e.g. confidence intervals)
☐	☒	For null hypothesis testing, the test statistic (e.g. *F*, *t*, *r*) with confidence intervals, effect sizes, degrees of freedom and *P* value noted<br>*Give P values as exact values whenever suitable.*
☒	☐	For Bayesian analysis, information on the choice of priors and Markov chain Monte Carlo settings
☒	☐	For hierarchical and complex designs, identification of the appropriate level for tests and full reporting of outcomes
☒	☐	Estimates of effect sizes (e.g. Cohen's *d*, Pearson's *r*), indicating how they were calculated

Our web collection on statistics for biologists contains articles on many of the points above.

Software and code

Policy information about availability of computer code

Data collection	Immunofluorescence images acquired using LASAF software, Flow cytometry data acquired using DIVA software (BD), Harmony (Perkin Elmer) used to collect live cell imaging data and immunofluroescence images for EdU staining.
Data analysis	Immunofluorescence images were analysed using ImageJ v1.52i and Flow cytometry data using FlowJo v10.0.8. Columbus software used to analyse EdU immunofluroescence data. NucliTrack (Barr et al. 2017) used to analyse live cell imaging data. Prism 8 for data display and statistical analysis

For manuscripts utilizing custom algorithms or software that are central to the research but not yet described in published literature, software must be made available to editors/reviewers. We strongly encourage code deposition in a community repository (e.g. GitHub). See the Nature Research guidelines for submitting code & software for further information.

Data

Policy information about availability of data

All manuscripts must include a data availability statement. This statement should provide the following information, where applicable:

- Accession codes, unique identifiers, or web links for publicly available datasets
- A list of figures that have associated raw data
- A description of any restrictions on data availability

The data in support of the findings of this study are available from the corresponding author upon reasonable request.

Field-specific reporting

Please select the one below that is the best fit for your research. If you are not sure, read the appropriate sections before making your selection.

☒ Life sciences ☐ Behavioural & social sciences ☐ Ecological, evolutionary & environmental sciences

For a reference copy of the document with all sections, see nature.com/documents/nr-reporting-summary-flat.pdf

Life sciences study design

All studies must disclose on these points even when the disclosure is negative.

Sample size	For DNA fibre analysis sample size was determined using published results Techer et al 2013. Other sample sizes were based on previously reported data
Data exclusions	Flow cytometry data was excluded based on the gating strategy described. Otherwise there were no data exclusions
Replication	Fig1c, 2a,b,c, Fig3b,c,d, Fig 4a, Supp Fig 1a, b, Supp Fig 2a,c-h, Supp Fig 3a,c,d, Supp Fig 4a,b n=3 experimental repeats, Fig 1f,g, Fig 3a, Fig 4b-d, Supp Fig 1d,e, Supp Fig 3b, Supp Fig 4c-e n=2 experimental repeats. MCM2 data in Supp Fig 2a was not repeated as the same result had already been shown using the MCM7 antibody.
Randomization	This is not relevant to our study, the cell lines and conditions used are specified in the text.
Blinding	Blinding was not always used as most analysis was done computationally using the same parameters and analysis methods (scripts and Macros) across conditions.

Reporting for specific materials, systems and methods

We require information from authors about some types of materials, experimental systems and methods used in many studies. Here, indicate whether each material, system or method listed is relevant to your study. If you are not sure if a list item applies to your research, read the appropriate section before selecting a response.

Materials & experimental systems

n/a	Involved in the study	
☐	☒	Antibodies
☐	☒	Eukaryotic cell lines
☒	☐	Palaeontology
☒	☐	Animals and other organisms
☒	☐	Human research participants
☒	☐	Clinical data

Methods

n/a	Involved in the study	
☒	☐	ChIP-seq
☐	☒	Flow cytometry
☒	☐	MRI-based neuroimaging

Antibodies

Antibodies used	RPA32 (RPA2) EMD Millipore MABE285; Phospho Histone H2A.X Ser139 Cell Signalling Technology, 9718S, 20E3; Rat anti-BrdU Bio-Rad Antibodies, OBT0030G; Mouse anti-BrdU, BD Biosciences, 347580; PCNA PC10 sc-56, Bethyl Laboratories; MCM2 sc9839, Bethyl Laboratories; MCM7 sc56324, Santa Cruz; Cdc6 sc9964, Santa Cruz; Cdc7 sc56274 Santa Cruz; Cyclin A sc53227, Santa Cruz; PCNA sc56291, Santa Cruz; GAPDH GT239, GeneTex; E2F6 ab155978 Abcam; Goat anti-Mouse IgG (H+L) Alexa Fluor 488 Life Technologies, A11029; Goat anti-rabbit IgG (H+L) Alexa Fluor 647 Life Technologies, A21244; Goat anti-rat Alexa Fluor 555 Life Technologies, A21434;
Validation	The antibodies are commercially available and already validated in the field. The use of positive and negative controls in the experiments is used to assess their reliability.

Eukaryotic cell lines

Policy information about cell lines

Cell line source(s)	RPE1 hTERT cells were provide by Horizon. The RPE1 hTERT p21GFP PCNA mRuby were kindly provided by Alexis Barr and are already published, Heldt et al 2018. The RPE1 hTERT TRex E2F6 were published Bertoli et al 2016.
Authentication	RPE1 hTERT cells from Horizon were authenticated, the other cell lines were not.
Mycoplasma contamination	We completed mycoplasma tests which were negative.

Commonly misidentified lines
(See ICLAC register)

Name any commonly misidentified cell lines used in the study and provide a rationale for their use.

Flow Cytometry

Plots

Confirm that:

☐ The axis labels state the marker and fluorochrome used (e.g. CD4-FITC).

☒ The axis scales are clearly visible. Include numbers along axes only for bottom left plot of group (a 'group' is an analysis of identical markers).

☒ All plots are contour plots with outliers or pseudocolor plots.

☒ A numerical value for number of cells or percentage (with statistics) is provided.

Methodology

Sample preparation

Flow cytometry was performed as described previously for DAPI/PCNA/MCM2/MCM7/EdU in Moreno et al 2016. Cells were incubated with 10 μM EdU for 30 min before trypsinisation and collection. Cells were pre-extracted in CSK buffer for 10 min on ice before fixation in 4% formaldehyde for 10 min at RT. Cells were permeabilised in ice cold 70% ethanol before incubation for 1 h at RT in primary antibody followed by secondary antibody. EdU was then detected using the Click iT-EdU assay (C10634 LifeTechnologies) according to the manufacturer's instructions and cells were treated with DAPI (0.5 μg/ml). Cells used were human retinal pigment epithelial cells RPE1 hTERT.

Instrument

BD LSRII

Software

DIVA Software (BD) used to collect the data and FlowJo software used for analysis

Cell population abundance

No FACS sorting was completed, so no populations selected.

Gating strategy

Live cells were gated using forward and side scatter. Single cells were gated according to peak area and height. Populations in Fig2b, 3d and Supp 2 were defined as follows: G1: EdU negative and G1 DNA content, Early S: EdU positive and G1 DNA content, Mid S: EdU positive and intermediate DNA content, Late S: EdU positive and G2 DNA content, G2: EdU negative and G2 DNA content.

☒ Tick this box to confirm that a figure exemplifying the gating strategy is provided in the Supplementary Information.
